# Pheromonal Regulation of the Reproductive Division of Labor in Social Insects

**DOI:** 10.3389/fcell.2020.00837

**Published:** 2020-08-20

**Authors:** Jin Ge, Zhuxi Ge, Dan Zhu, Xianhui Wang

**Affiliations:** ^1^State Key Laboratory of Integrated Management of Pest Insects and Rodents, Institute of Zoology, Chinese Academy of Sciences, Beijing, China; ^2^CAS Center for Excellence in Biotic Interactions, University of Chinese Academy of Sciences, Beijing, China

**Keywords:** division of labor, queen pheromone, juvenile hormone, olfactory receptor, caste

## Abstract

The reproductive altruism in social insects is an evolutionary enigma that has been puzzling scientists starting from Darwin. Unraveling how reproductive skew emerges and maintains is crucial to understand the reproductive altruism involved in the consequent division of labor. The regulation of adult worker reproduction involves conspecific inhibitory signals, which are thought to be chemical signals by numerous studies. Despite the primary identification of few chemical ligands, the action modes of primer pheromones that regulate reproduction and their molecular causes and effects remain challenging. Here, these questions were elucidated by comprehensively reviewing recent advances. The coordination with other modalities of queen pheromones (QPs) and its context-dependent manner to suppress worker reproduction were discussed under the vast variation and plasticity of reproduction during colony development and across taxa. In addition to the effect of QPs, special attention was paid to recent studies revealing the regulatory effect of brood pheromones. Considering the correlation between pheromone and hormone, this study focused on the production and perception of pheromones under the endocrine control and highlighted the pivotal roles of nutrition-related pathways. The novel chemicals and gene pathways discovered by recent works provide new insights into the understanding of social regulation of reproductive division of labor in insects.

## Introduction

Insect societies provide excellent model systems for research on organization principles. A signature and defining trait of eusocial insects is the reproductive division of labor, expressed as strong reproductive skew, in which a single or a few females (queen) monopolize colony reproduction, while all other females (workers) care for eggs laid by the queen (Wilson, 1971). Unraveling the mechanism underlying reproductive skew provides an avenue to understand the influence of social interaction on individual phenotypic plasticity and its consequent task allocation.

Social insects are well known to utilize chemical signals to regulate their behavior (Leonhardt et al., 2016). Empirical evidence and theoretical consideration indicated the importance of pheromones in regulating adult worker reproduction. These pheromones, predicted by classical paradigms, are specific components to dominant females [queen pheromones (QPs)] and are closely correlated with fertility. On the basis of this criterion, early studies have identified queen mandibular pheromones (QMPs) in honeybees and cuticular hydrocarbons (CHCs) in ants as queen primer pheromones to inhibit worker reproduction (Le Conte and Hefetz, 2008). However, two questions remain unsettled, including (1) the modes of action of chemical components and (2) the pheromonal production and the modulation of pheromones on worker physiology.

This study aimed to review research progresses focusing on the above two questions mainly in the last 10 years, from ultimate and proximate perspective. The influence of context and the coordination with other modalities in reproductive regulation were discussed under the complexity of reproductive skew at colony levels. In addition to QPs, the effect of brood was considered. Given the strong association between pheromone and hormone, we focused on the production and perception of pheromones in response to endocrine factors. Taking advantage of exquisite bioassays, chemical analysis, genomics, and genetic manipulation, recent discoveries have found molecular innovation of chemicals and gene pathways, largely expanding the understanding of social regulation of reproduction in insects.

## Reproductive Signaling: a Combination of Sensory Modalities

The modalities that queens or foundresses utilize to regulate workers could be generally categorized into either “behavioral” or “chemical.” In primitive eusocial species lacking morphologically defined castes, aggressive dominance behavior is a universal approach to generate reproductive skew and could be aided by visual cues serving as “badges of status” (Tibbetts and Lindsay, 2008). By contrast, in highly social species with a large colony, queens were unable to afford physical contact with every worker. Therefore, chemical communication is hypothesized to be a reliable way to regulate worker reproduction (Le Conte and Hefetz, 2008; Kocher and Grozinger, 2011). Despite a correlation between CHCs and fertility of dominant breeders in many primitively eusocial wasps (Sledge et al., 2001; Dapporto et al., 2007; Bhadra et al., 2010), the queen CHC blends alone failed to inhibit the subordinates’ ovarian development in *Polistes satan*. Therefore, these studies supported the hypothesis that fertility-linked compounds only play addictive roles in the reproductive regulation of primitively eusocial species (Oi et al., 2019). However, a recent study in *Lasioglossum malachurum* sweat bees has revealed that the over production of macrocyclic lactones acts as QPs by influencing worker behavior and decreasing ovarian activations. This study for the first time described a QP with a primer function in a eusocial hymenopteran species other than species with morphologically distinct castes (Steitz and Ayasse, 2020). Similar to the complexity of modalities regulating reproduction in primitively eusocial species, mounting evidence of QPs in highly eusocial species did not exclude the participation of behavioral interaction in affecting worker reproduction. In *Bombus impatiens* bumblebees, directly contacting with a caged queen could fully activate worker ovaries, suggesting that queen-initiated behavior may be responsible for the inhibition of worker reproduction (Padilla et al., 2016). Taken together, the orchestration of pheromone and behavior in regulating worker reproduction seem more complex than previously conceived. Given that the transmission of each sensory modality is under the selection of environmental factors, reproductive signaling is possibly affected by species-specific life-history traits. In the case of sweat bees, living in below-ground nests renders visual cues useless and is likely to make chemical compounds as an honest signal of dominance. How various modalities (i.e., behavior, chemical compounds and visual cues) interact to regulate reproduction remains an open question. Finally, the establishment of reproductive hierarchy requires the cognition of nestmates’ fertility which integrates various sensory modalities, such as behavior, chemicals and visual cues. The outcome of behavioral interaction could be inferred by the color pattern or chemical fingerprints of opponents (Reichert and Quinn, 2017), thus eliciting associative learning after dominance contest.

In addition to numerous works focusing on reproductive signaling from queens and nestmate workers, recent studies have examined the roles of brood in reproductive regulation. When kept with young larvae, *B. impatiens* workers showed reduced ovarian activation and oviposition. Moreover, this effect was in a quantity-dependent manner; the presence of 10 larvae completely suppresses egg laying, regardless of worker age, relatedness to brood, or brood parentage/sex (Starkey et al., 2019a). Exposure to the odors from larvae, however, was insufficient to suppress ovarian activation and workers fail to differentiate between larvae and pupae on the basis of olfactory cues (Starkey et al., 2019b). Therefore, in bumblebees, odors alone could not explain regulatory effect of brood and workers might use multiple information sources or rely on behavioral interaction to regulate their reproduction. A subsequent study further showed a synergistic effect of queens and larvae on worker reproduction-related genes, where the combined effect of queen and larvae outweighed their separate effect, indicating that the two coordinate to maintain reproductive monopoly (Orlova et al., 2020). The regulatory effect of young offspring, including eggs, is likely to evolve across social species, given the widespread trade-off between reproduction and brood care (Schultner et al., 2017). In highly eusocial *Apis mellifera* and primitively eusocial *Ooceraea biroi*, the worker’s ovary activation is suppressed by larvae signals which are identified as chemical compound in honeybees (Mohammedi et al., 1998; Maisonnasse et al., 2010; Ulrich et al., 2016). Eggs could also convey inhibitory signals to workers in a highly eusocial insect; the surface hydrocarbons of *Camponotus floridanus* queen-laid eggs induce workers to refrain from oviposition, thus regulating worker reproduction in subcolonies (Endler et al., 2004).

## Evolutionary Origin of QPs

Two scenarios regarding the evolutionary origin of QPs prevail. Widely discussed and supported in CHC QPs is the sender-precursor scenario. This hypothesis posits that QPs were derived from a preadaptation on the sender, who already produces a precursor as functionless physiological byproducts or to serve unrelated purpose (Wyatt, 2014; Stokl and Steiger, 2017). Under this scenario, two predictions could be proposed. The first one is that QPs evolve from chemical cues of solitary ancestors, as byproducts of ovary development. This hypothesis is robustly supported by the universal physiological link between odor and fertility (Van Oystaeyen et al., 2014; Holman, 2018). In solitary and primitive eusocial species without morphologically distinct castes, ovarian activation usually triggers substantial changes in cuticular compounds (Liebig, 2010; Oi et al., 2019). Thus, the chemical by-products of fertility gradually evolved into honest fertility indices and subsequently into dedicated QPs in highly eusocial species (Peeters and Liebig, 2009). Even though widely accepted as one of the most convincing explanations regarding QP evolution, the mechanisms underlying functional transition from fertility recognition cues to ovarian developmental deterrents remain elusive. The second prediction is that QPs may be derived from sex pheromones. From an ultimate perspective, sex pheromones advertise female fecundity to potential mates and thus are able to serve as honest indicators of fertility and evolve secondarily as QPs (Ayasse et al., 2001). A support for this prediction came from the shared structure between fertility signals in social species and contact pheromones in solitary species (Smith et al., 2009, 2012). However, limited studies have demonstrated a joint function in a certain species. The only evidence comes from honeybees in which chemicals from QMPs not only attract males from long distance but also induce worker sterility (Le Conte and Hefetz, 2008). The evolution of QP’s dual functions is possibly constrained by the life-cycle separation between mating and ovarian activation. For instance, in the sweat bee, gynes mate before overwintering but initiate reproduction in the ensuing summer. Sex pheromones are not used as QPs, because of their quantitative decrease after mating (Steitz and Ayasse, 2020).

In contrast to the sender–precursor scenario, the sensory exploitation hypothesis predicted that QPs could evolve *de novo* acting on pre-existing gene-regulatory networks that are linked with the regulation of reproduction in receiver workers. A notable case under this scenario is the honeybee QMPs. On the one hand, the 4-hydroxy-3-methoxyphenylethanol (HVA) in QMPs could directly suppress the worker reproduction by acting on dopamine receptors instead of stimulating the sensory system (Beggs et al., 2007; Beggs and Mercer, 2009). On the other hand, the QMPs unexpectedly showed reduced ovary size, number of eggs and the number of viable offspring in phylogenetically distantly related fruit fly *Drosophila melanogaster*, suggesting the exploitation of conserved physiological pathways (Camiletti et al., 2013; Galang et al., 2019). This finding was further verified by a recent study demonstrating a remarkable cross activity of honeybee QMP in bumblebees, in which the egg laying of workers and queens are inhibited by non-native QMP blend (Princen et al., 2019). Another hypothesis under the sensory exploitation scenario is that QPs evolve from oviposition deterring pheromones. In solitary insects, especially herbivores, females reduce egg laying to avoid intra-specific competition in response to conspecific chemical signals deposited on oviposition substrates (Peter, 2002). However, this hypothesis lacks any empirical evidence.

The two above-mentioned scenarios are in line with the two hypotheses regarding the ultimate cause of the reproductive skew in social insects. In particular, the sender-precursor scenario corresponds to the queen signal hypothesis which postulates that workers regulate their own reproduction on the basis of their own fitness interest thus adjusting their behavior in accordance to queen signals that honestly indicate queen fertility (Keller and Nonacs, 1993; Grueter and Keller, 2016). By contrast, under the “sensory exploitation” scenario, which corresponds to “queen control” hypothesis, the queen could potentially deceive or manipulate the workers against their own interests even though the signals are still honest (Keller, 2009; Smith and Liebig, 2017). This sensory exploitation could be evolutionarily stable in the long run, by leading to either a queen–worker arm race, to workers evolving counter–adaptations, or to limited personal cost of workers’ sterility (Kocher and Grozinger, 2011; Peso et al., 2015). From a proximate perspective, the evolution of manipulative QPs requires the queens to be immune to the pheromones themselves, considering the inhibitory effect of honeybee QMP on bumblebee queens (Princen et al., 2019).

## Chemical Nature of Pheromones

**FIGURE 1:**
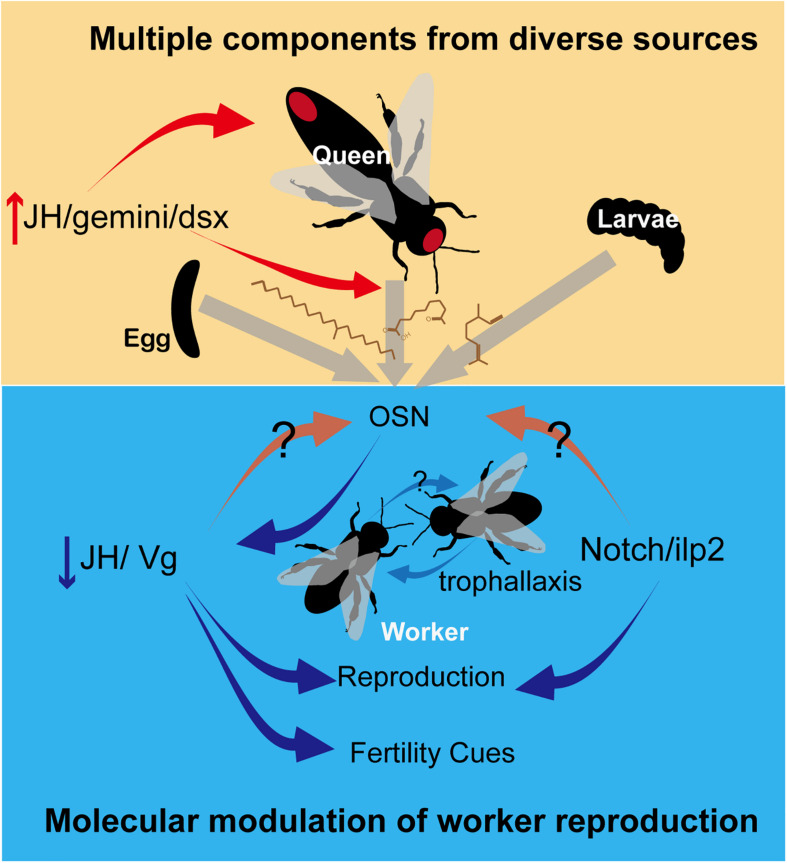
Pheromonal regulation of worker reproduction in social insects. Primer pheromones, including CHCs and other glandular compounds, are released by queens, eggs and larvae to suppress worker ovarian development. The production of pheromones is influenced by gonadotropin JH or by transcriptional factor gemini or dsx. In recipient workers, the primer pheromones down-regulate nutrition-related pathways including JH/Vg or Notch/ilp to inhibit worker reproduction, via activating OSN in antennae. These nutrition-related pathways may increase the sensitivity of OSN to pheromones in turn. An amplification of effect is possible due to trophallaxis among workers. Moreover, a down-regulated JH level results in a reduction of fertility cues emitted by workers, therefore maintaining reproductive skew in a colony. Red ellipses in the upper panel show glands in queens. Red and blue arrows depict stimulatory and inhibitory effects, respectively.

Studies in recent years have focused on the role of CHCs as QPs. Synthesized in oenocytes, the arrays of long-chained linear and branched, saturated, and unsaturated hydrocarbons are highly abundant in insect epicuticle, conveying recognition cues related to sex, age, and nestmates (Oi et al., 2015). Mounting evidence showed that CHCs are conserved honest fertility cues, from primitively eusocial wasps to advanced eusocial ants (Liebig, 2010). A recent study identified CHC as a royal pheromone in termites, which appears to predate its use as QP in social Hymenoptera (Funaro et al., 2018). However, the unequivocal identification of CHCs with priming function is limited in a handful of species, thus preventing the generalization of the chemical nature of QP. The inhibitory effect of isolated CHC signal on ovarian activation were observed in ants, wasps, and bumblebees, implying that CHCs are a conserved class of signals in regulating reproduction (Van Oystaeyen et al., 2014). However, the uniformity of CHCs as QPs is challenged by subsequent studies regarding its methodological bias and logical pitfall. First, because of CHCs abundance and ease in detection and spectral/structural analysis, the research preference for CHCs inevitably caused negligence of other compounds (Amsalem and Hefetz, 2018). Therefore, recent reviews and studies called for attention to be paid to the exocrine glands, which are the sources of many described trial and alarm pheromones (Dani and Turillazzi, 2018; Villalta et al., 2018; Hefetz, 2019). Up to date, the only QP found in exocrine glands is the chemical blends in the honeybee mandibular glands, which are immensely hypertrophied in queens (Slessor et al., 1988). Indeed, the queen-specific specialization has been reported in various glands and species, such as honeybees (Katzav-Gozansky et al., 1997), halictid bees (Steitz and Ayasse, 2020), and ants (Smith et al., 2012), but dedicated examination of their compounds functioning as QPs has been seldom performed. Second, although non-volatile CHCs are able to avoid sensory habituation caused by saturation, their higher expression in queens compared with workers cannot guarantee the uniqueness of QPs predicted by theory (Hefetz, 2019). Most of the identified CHC QPs are common in queens and workers but present in greatest quantities in queens (Van Oystaeyen et al., 2014). Regulation via the QP higher in queens than in workers could be problematic because of a dilution of pheromones in populous colonies (Orlova and Hefetz, 2014). Workers possibly activate their ovaries as under queenless conditions. Overall, despite the certain roles of CHCs in adverting the queen’s presence, these fertility cues are not sufficient to serve as conserved signals for the regulatory function in reproduction. Glandular sources and other queen-qualitatively specific compounds should be considered in the search for queen primer pheromones (Figure 1).

Recent studies and reviews have proposed several guidelines in seeking unknown QPs (Amsalem and Hefetz, 2018; Villalta et al., 2018; Hefetz, 2019). Firstly, QPs may be multicomponent and pleiotropic. The specificity of communication is theoretically enhanced when compound blends are used as pheromones. Multiple components may contribute to the reliability of queen signaling because of the complexity of social signals within a colony. In honeybees, the QPs consists of six components, all of which are necessary for the inhibition of gyne rearing (Wanner et al., 2007; Matsuura et al., 2010). Besides, QPs could also elicit releaser effects, such as retinue behavior in ants and honeybee workers (Villalta et al., 2018), grooming in wasps (Holman et al., 2010), and shaking in termites (Funaro et al., 2018). Secondly, the QPs usually work in a context dependent manner to prevent misinterpretation (Orlova and Amsalem, 2019). A growing body of evidence has identified various forms of social context responsible for the expression of QP regulatory effects, including chemical background (Funaro et al., 2018), behavioral repertoire (Padilla et al., 2016), or the presence of brood (Hoover et al., 2003) or nestmates (Kikuchi et al., 2007). Social context may influence QP dispersion within the colony. For example, in honeybees and ants, the frequent occurrence of licking or trophallaxis possibly helps to transfer QP and accelerate the dispersion of QP from queen to workers and among workers (Naumann et al., 1991; Soroker et al., 1995). In addition, thermal regulation by workers is an alternative way to facilitate QP dispersion. The capacity of honeybee and bumble workers to produce heat may promote the active space of QPs (Stabentheiner et al., 2010), which generally increase QP volatility as temperature increases (Gibbs, 1995).

## Pheromonal Production

The production of QPs in queens is still mysterious in most social insect species, but it could be inferred by their link with reproduction. A large body of studies have revealed the role of juvenile hormone in the production of CHCs. The linkage between CHCs and fertility in social insects could be explained by the universal hormone pleiotropy reported in primitively and advanced eusocial species, in which Juvenile Hormone (JH) serves as CHC regulator and gonadotropin hormone (Slessor et al., 1988; Cuvillier-Hot et al., 2002; Holman et al., 2010; Kelstrup et al., 2014; Oliveira et al., 2017). The up-regulation of JH via the application of methoprene (a JH analog) leads to a shift of worker CHC profile to that of the queen (Brent et al., 2016; Oliveira et al., 2017). Thus, the hormones involved in ovarian development could affect the synthesis or transport of CHCs. However, the genes responsible for the synthesis of CHC fertility signal in social insects have only been reported in termites. *Neofem4*, a cytochrome P450 gene, is more expressed in *Cryptotermes secundus* queens than in workers. Silencing this gene by using RNAi alters the queen odors to those of a worker and induces a worker butting behavior, indicating the onset of reproductive replacement differentiation (Korb et al., 2009; Hoffmann et al., 2014). However, less information is available regarding the production of QP in exocrine glands. In Cape honeybees, the synthesis of QMPs is controlled by alternative splicing of the CP-2 transcription factor *gemini*. Altering the splice pattern of *gemini* by siRNA feeding results in ovary activation and increased amount of queen-specific pheromones 9-oxo-2-decenoic acid (9-ODA) and 9-hydroxy-2-decenoic acid (9-HDA). As methyl p-hydroxybenzoate (HOB) and HVA are not affected by the knockdown of specific *gemini*, this gene possibly acts on the biosynthesis of fatty acid-derived components (Jarosch et al., 2011; Jarosch-Perlow et al., 2018). The synthesis of QMP is also influenced by the sex determination pathway. The knockdown of *Doublesex (dsx)* in queenless honeybee workers results in reduced ovary development and a reduced level of pheromonal fertility signals, indicating that the regulatory network is co-opted during eusocial evolution to regulate pheromone production (Velasque et al., 2018). Considering the honesty of QPs in indicating fertility predicted by the sender–precursor scenario (see section “EVOLUTIONARY ORIGIN OF QPS”), gonadotropin may be pivotal in the production of QPs besides CHCs. In addition to gonadotropin, nutrition is likely to be an alternative proximate factor governing QP production, because it activates reproduction directly on vitellogenin (vg) or indirectly through JH (Kapheim, 2017). Nutrition is also the key to influence reproductive physiology and drives caste determination in honeybees (Metcalf and Whitt, 1977; Mutti et al., 2011).

## Pheromonal Perception

A key issue regarding pheromone-regulated reproduction is how QPs are perceived and what sort of genetic and physiological specializations facilitate signal perception in social insects. Semio-chemicals are basically detected by olfactory sensory neurons in porous sensory hairs located on chemosensory organs, especially the antennae. The knock-down of Orco, an olfactory co-receptor, impairs a series of social behavior including worker dueling to become gametes in *Harpegnathos saltator* ants (Yan et al., 2017). Electroantennography demonstrated that workers respond specifically to the putative QPs in several species, such as *Pachycondyla inversa*, *Apis cerana*, and *A. mellifera* (D’Ettorre et al., 2004; Dong et al., 2017). Moreover, single sensilla recording in ants verified that QP candidate CHCs are responded by basiconic sensilla (Ozaki et al., 2005; Sharma et al., 2015). Investigations of the glomeruli in the antennal lobe where ORNs are projected to in the brain have revealed female-specific or caste-specific structural specialization relevant to QP perception. In *C. floridanus*, females have about approximately 140 T6 glomeruli, whereas males completely lack these glomeruli (Nakanishi et al., 2010). In *O. biroi*, basiconic sensilla and CHC responsive ORs are found only on the ventral surface of the female antennal club (McKenzie et al., 2016). A correlation between glomeruli numbers and worker polymorphism exists in *Atta vollenweideri* (Kelber et al., 2010). Given their central role in chemosensory perception, peripheral circuits substantially contribute to the specificity and plasticity of QP perception. For instance, *C. floridanus* workers are able to detect and distinguish enantiomers of a proposed QP (3-methylheptacosane) by using sensilla basiconica structures (Sharma et al., 2015). Electrophysiological responses to several CHCs decrease with the transition from non-reproductive *H. saltator* workers to gamergates, implying a potential mechanism to tolerate the mutually inhibitory or self-inhibitory effects of QPs on gamergates (Gospocic et al., 2017).

Recent works have implied possible correlation between pheromonal perception and endocrine factors. In honeybees, JH treatment influences the retinue behavior of young workers to QMP. The latter influence is likely due to the reduced levels of an octopamine (OA) receptor in the antennae (Vergoz et al., 2009). Indeed, JH application induces a dramatic change in gene expression in the neural system, including the brain and antennae (Pandey and Bloch, 2015). A similar modulation of responsiveness to social cues has also been reported in Vg. A knockdown of a Vg ortholog called *Vg-like A* in the fat body of young ant workers reduced brood care and increased nestmate care, a task usually performed by old workers. This work revealed that *Vg-like A* drives behavioral maturation by mediating responses to social cues (Kohlmeier et al., 2018). Thus, divergent Vg or JH titer may lead to differential responses to QPs among castes. Further study should investigate the mechanisms of sensory threshold modulation by endocrine factors in primitively and advanced eusocial species.

Analysis based on genome has revealed that 9-exon ORs particularly expanded in ants are specifically expressed in females, indicating a possible interaction with social signals, such as CHCs (Smith et al., 2011, 2012; Zhou et al., 2012; Oxley et al., 2014; McKenzie et al., 2016). The knowledge of OR with ligands in QPs is limited in two species. In honeybees, AmOr11, specifically bounds to the QMP component 9-ODA (Wanner et al., 2007). In *H. saltator*, HsOr263 is highly expressed in workers and responsible in binding a candidate reproductive signal, due to its strong responses to gamergate extract and a predicted QP (13, 23-DiMeC37) (Pask et al., 2017). Whether and how the olfactory plasticity of QP perception is influenced by modulation of OR expression levels remain to be determined, because alterations in a single odorant receptor may lead to a significant effect on animal odor perception.

## Pheromonal Effects at Molecular Levels

Few studies have revealed the global effect of QPs on the genetic and epigenetic networks of workers. In *Solenopsis invicta*, under queenright conditions, numerous genes are differentially expressed between foragers and non-foragers. However, in absence of the queen, these differences disappear at transcriptional level, implying a possibility that QP participates in task allocation among workers (Manfredini et al., 2014). A comparative study further showed that exposure to QPs leads to similar trancriptic responses in two ant and two bee species (genera: *Lasius*, *Apis*, and *Bombus*). With functions involving lipid biosynthesis and transport, olfaction, production of cuticle, oogenesis, and histone (de) acetylation, QP-sensitive genes seem to be peripheral in the gene co-expression network and caste-specific expression (Holman et al., 2019). In addition to the effect of QP on gene expression, the application of QP results in a change in the level of DNA methylation in honeybee and *Lasius* ants. However, the DNA methylation level is not affected by QP in *Bombus terrestris* (Holman et al., 2016). The QMP in honeybees also exerts epigenetic modifications to RNA and histones in the brain, potentially affecting aging-related genes (Junior et al., 2020). Although promising, these findings mainly examined the global level of epigenetic effect, while the mechanism of QP perception on epigenetics and epigenetically targeted genes remain unknown.

Studies concerning the specific molecular effects of reproductive pheromonal regulation in adult individuals are scarce. Duncan et al. (2016) provided the first molecular mechanism directly linking ovary activity in adult worker bees with the presence of the queen. QMP inhibits worker reproduction by stimulating Notch signaling in ovary tissues in the region where germ cells are specified. In the absence of the queen, the Notch receptor in the cells of germarium renders these cells refractory to Notch signaling. This finding provided support for the reproductive ground plan hypothesis (West-Eberhard, 1987; Page and Amdam, 2007), by revealing the targeted control of worker fertility through co-option of a conserved cell-signaling pathway (Duncan et al., 2016). In addition to the Notch, alternative pathways have been proposed in primitively eusocial species. For example, in *B. terrestris*, Vg expression is lower in the presence of newly mated queens than same-age virgin queens regardless of queen ovarian inactivation under both conditions (Amsalem et al., 2014). In *Polistes dominula* paper wasps, 3-h group formation is sufficient to down-regulate the JH level in low-ranked subordinates, suggesting the hyper-sensitivity of JH in response to nestmate cues, including pheromones (Tibbetts et al., 2018). This result was reminiscent of the finding that JH could mediate the threshold to social signals in turn. Given the gonadotropic and physiological functions of JH in the primitively social species (Amsalem et al., 2014; Kelstrup et al., 2017), a negative feedback loop exists between pheromones and JH to enhance the efficiency of regulation (Figure 1). Recent studies on parthenogenetic clonal raider ants (*O. biroi*) revealed the molecular mechanism underlying the brood control of reproductive skew. A single gene, called insulin-like peptide 2 (*ilp2*) was identified on the basis of brain transcriptome. This gene was more up-regulated in reproductives than in non-reproductives (Chandra et al., 2018). Larval signals suppress *ilp2* to inhibit adult reproduction, thus potentially amplifying reproductive asymmetries (Chandra et al., 2018). These studies have partially revealed the signal transduction from social cues, including pheromones to reproductive outcome, and highlighted the importance of nutrition-related pathways. Whether pheromones directly affect the ovary or act via signaling between neural circuits are yet to be determined. Although ovarian development was extensively used as a proxy to reproduction, ovarian activation may be decoupled from reproduction because the ovary is possibly involved in the task allocation of workers, as demonstrated by the meta-analysis in some ant species (Pamminger and Hughes, 2017). Further studies should pay attention to the functional discrimination between ovarian activation and egg laying in reproduction.

## Conclusion

Considerable advances on the pheromonal regulation of reproduction have been achieved in the last decades. Several discoveries of variation and plasticity of reproductive partitioning within and across species provided not only valuable data for constructing evolutionary routes of chemical communication but also hints for the identification of key components via a comparative approach.

Despite the strong association between CHCs and fertility, the unequivocal identification of CHC as queen pheromone. QP is rather limited. The contexts, such as colony, nestmates, and brood, in the regulation of worker reproduction demonstrated that pheromones are released from more than one source with multiple components and should work in combination with other modalities (Figure 1). This argument helps settle the existing controversies among the chemical nature, evolutionary origin, and conservation of reproductive signaling.

Current works in both primitively eusocial and advanced eusocial species indicated the co-option of conserved pathways in pheromonal regulation of reproduction and highlighted the coordination among social cues, endocrine factors and reproduction (Figure 1). Studies on model species, such as honeybees and radial colonial ants, have demonstrated evolutionary innovations of nutrition-related pathways responsible for pheromonal effects. However, the mechanism of production and reception of pheromones remains largely unknown. With the aid of advances in genetic manipulation, future works could elucidate the genetic network underlying QP production, thereby contributing to loss-of-function experiments to identify the causal relationships between semio-chemicals and reproduction. The reverse chemical ecology, based on the genome and identification of the olfactory receptors will be an effective approach for the screening of new components.

## Author Contributions

XW and JG designed the research. JG and ZG collected the references. All authors wrote the manuscript.

## Conflict of Interest

The authors declare that the research was conducted in the absence of any commercial or financial relationships that could be construed as a potential conflict of interest.

## References

[B1] Amsalem E. Hefetz A. (2018). Preface: pheromone-mediation of female reproduction and reproductive dominance in social species. *J. Chem. Ecol.* 44 747–749. 10.1007/s10886-018-0992-7 30009328

[B2] Amsalem E. Malka O. Grozinger C. Hefetz A. (2014). Exploring the role of juvenile hormone and vitellogenin in reproduction and social behavior in bumble bees. *BMC Evol. Biol.* 14:45. 10.1186/1471-2148-14-45 24618396 PMC4007805

[B3] Ayasse M. Paxton R. Tengö J. (2001). Mating behavior and chemical communication in the order hymenoptera. *Annu. Rev. Entomol.* 46 31–78. 10.1146/annurev.ento.46.1.31 11112163

[B4] Beggs K. T. Glendining K. A. Marechal N. M. Vergoz V. Nakamura I. Slessor K. N. (2007). Queen pheromone modulates brain dopamine function in worker honey bees. *Proc. Natl. Acad. Sci. U.S.A.* 104 2460–2464. 10.1073/pnas.0608224104 17287354 PMC1892986

[B5] Beggs K. T. Mercer A. R. (2009). Dopamine receptor activation by honey bee queen pheromone. *Curr. Biol.* 19 1206–1209. 10.1016/j.cub.2009.05.051 19523830

[B6] Bhadra A. Mitra A. Deshpande S. A. Chandrasekhar K. Naik D. G. Hefetz A. (2010). Regulation of reproduction in the primitively eusocial wasp *Ropalidia marginata*: on the trail of the queen pheromone. *J. Chem. Ecol.* 36 424–431. 10.1007/s10886-010-9770-x 20238237

[B7] Brent C. S. Penick C. A. Trobaugh B. Moore D. Liebig J. (2016). Induction of a reproductive-specific cuticular hydrocarbon profile by a juvenile hormone analog in the termite *Zootermopsis nevadensis*. *Chemoecology* 26 195–203. 10.1007/s00049-016-0219-8

[B8] Camiletti A. L. Percival-Smith A. Thompson G. J. (2013). Honey bee queen mandibular pheromone inhibits ovary development and fecundity in a fruit fly. *Entomo. Exp. Appl.* 147 262–268. 10.1111/eea.12071

[B9] Chandra V. Fetter-Pruneda I. Oxley P. R. Ritger A. L. McKenzie S. K. Libbrecht R. (2018). Social regulation of insulin signaling and the evolution of eusociality in ants. *Science* 361 398–402. 10.1126/science.aar5723 30049879 PMC6178808

[B10] Cuvillier-Hot V. Gadagkar R. Peeters C. Cobb M. (2002). Regulation of reproduction in a queenless ant: aggression, pheromones and reduction in conflict. *Proc. Biol. Sci.* 269 1295–1300. 10.1098/rspb.2002.1991 12065047 PMC1691022

[B11] Dani F. R. Turillazzi S. (2018). Chemical communication and reproduction partitioning in social wasps. *J. Chem. Ecol.* 44 796–804. 10.1007/s10886-018-0968-7 29785627

[B12] Dapporto L. Santini A. Dani F. R. Turillazzi S. (2007). Workers of a Polistes paper wasp detect the presence of their queen by chemical cues. *Chem. Senses* 32 795–802. 10.1093/chemse/bjm047 17644826

[B13] D’Ettorre P. Heinze J. Schulz C. Francke W. Ayasse M. (2004). Does she smell like a queen? Chemoreception of a cuticular hydrocarbon signal in the ant *Pachycondyla inversa*. *J. Exp. Biol.* 207 1085–1091. 10.1242/jeb.00865 14978051

[B14] Dong S. Wen P. Zhang Q. Li X. Tan K. Nieh J. (2017). Resisting majesty: *Apis cerana*, has lower antennal sensitivity and decreased attraction to queen mandibular pheromone than *Apis mellifera*. *Sci. Rep.* 7:44640. 10.1038/srep44640 28294146 PMC5353700

[B15] Duncan E. J. Hyink O. Dearden P. K. (2016). Notch signalling mediates reproductive constraint in the adult worker honeybee. *Nat. Commun.* 7:12427. 10.1038/ncomms12427 27485026 PMC4976197

[B16] Endler A. Liebig J. Schmitt T. Parker J. E. Jones G. R. Schreier P. (2004). Surface hydrocarbons of queen eggs regulate worker reproduction in a social insect. *Proc. Natl. Acad. Sci. U.S.A.* 101 2945–2950. 10.1073/pnas.0308447101 14993614 PMC365725

[B17] Funaro C. F. Boroczky K. Vargo E. L. Schal C. (2018). Identification of a queen and king recognition pheromone in the subterranean termite *Reticulitermes flavipes*. *Proc. Natl. Acad. Sci. U.S.A.* 115 3888–3893. 10.1073/pnas.1721419115 29555778 PMC5899469

[B18] Galang K. Croft J. Thompson G. Percival-Smith A. (2019). Analysis of the Drosophila melanogaster anti-ovarian response to honey bee queen mandibular pheromone. *Insect Mol. Biol.* 28 99–111. 10.1111/imb.12531 30159981

[B19] Gibbs A. (1995). Physical properties of insect cuticular hydrocarbons: model mixtures and lipid interactions. *Comp. Biochem. Phys. B* 112 667–672. 10.1016/0305-0491(95)00119-0

[B20] Gospocic J. Shields E. J. Glastad K. M. Lin Y. Penick C. A. Yan H. (2017). The *Neuropeptide corazonin* controls social behavior and caste identity in ants. *Cell* 170 748–759. 10.1016/j.cell.2017.07.014 28802044 PMC5564227

[B21] Grueter C. Keller L. (2016). Acoustical communication in social insects. *Curr. Opin. Neurobiol.* 38 6–11. 10.1007/978-3-0348-8878-3_10 26803006

[B22] Hefetz A. (2019). The critical role of primer pheromones in maintaining insect sociality. *Z. Naturforsch. C. J. Biosci.* 74 221–231. 10.1515/znc-2018-0224 30920959

[B23] Hoffmann K. Gowin J. Hartfelder K. Korb J. (2014). The scent of royalty: a p450 gene signals reproductive status in a social insect. *Mol. Biol. Evol.* 31 2689–2696. 10.1093/molbev/msu214 25053804

[B24] Holman L. (2018). Queen pheromones and reproductive division of labor: a meta-analysis. *Behav. Ecol.* 29 1199–1209. 10.1093/beheco/ary023

[B25] Holman L. Helantera H. Trontti K. Mikheyev A. S. (2019). Comparative transcriptomics of social insect queen pheromones. *Nat. Commun.* 10:1593. 10.1038/s41467-019-09567-2 30962449 PMC6453924

[B26] Holman L. Jørgensen C. G. Nielsen J. d’Ettorre P. (2010). Identification of an ant queen pheromone regulating worker sterility. *Proc. Biol. Sci.* 277 3793–3800. 10.1098/rspb.2010.0984 20591861 PMC2992706

[B27] Holman L. Trontti K. Helantera H. (2016). Queen pheromones modulate DNA methyltransferase activity in bee and ant workers. *Biol. Lett.* 12:20151038. 10.1098/rsbl.2015.1038 26814223 PMC4785935

[B28] Hoover S. E. Keeling C. I. Winston M. L. Slessor K. N. (2003). The effect of queen pheromones on worker honey bee ovary development. *Naturwissenschaften* 90 477–480. 10.1007/s00114-003-0462-z 14564409

[B29] Jarosch A. Stolle E. Crewe R. M. Moritz R. F. (2011). Alternative splicing of a single transcription factor drives selfish reproductive behavior in honeybee workers (*Apis mellifera*). *Proc. Natl. Acad. Sci. U.S.A.* 108 15282–15287. 10.1073/pnas.1109343108 21896748 PMC3174643

[B30] Jarosch-Perlow A. Yusuf A. A. Pirk C. W. W. Crewe R. M. Moritz R. F. A. (2018). Control of mandibular gland pheromone synthesis by alternative splicing of the CP-2 transcription factor gemini in honeybees (*Apis mellifera* carnica). *Apidologie* 49 450–458. 10.1007/s13592-018-0571-5

[B31] Junior C. A. C. Ronai I. Hartfelder K. Oldroyd B. P. (2020). Queen pheromone modulates the expression of epigenetic modifier genes in the brain of honey bee workers. *bioRxiv* [Preprint], 10.1101/2020.03.04.977058 PMC7775979 33290662

[B32] Kapheim K. M. (2017). Nutritional, endocrine, and social influences on reproductive physiology at the origins of social behavior. *Curr. Opin. Insect Sci.* 22 62–70. 10.1016/j.cois.2017.05.018 28805640

[B33] Katzav-Gozansky T. Soroker V. Hefetz A. Cojocaru M. Erdmann D. Francke W. (1997). Plasticity of caste-specific Dufour’s gland secretion in the honey bee (*Apis mellifera* L.). *Naturwissenschaften* 84 238–241. 10.1007/s001140050386

[B34] Kelber C. Rossler W. Kleineidam C. J. (2010). Phenotypic plasticity in number of glomeruli and sensory innervation of the antennal lobe in leaf-cutting ant workers (*A. vollenweideri*). *Dev. Neurobiol.* 70 222–234. 10.1002/dneu.20782 20029932

[B35] Keller L. (2009). Adaptation and the genetics of social behaviour. *Philos. T. R. Soc. B* 364 3209–3216. 10.1098/rstb.2009.0108 19805428 PMC2781871

[B36] Keller L. Nonacs P. (1993). The role of queen pheromones in social insects: queen control or queen signal? *Anim. Behav.* 45 787–794. 10.1006/anbe.1993.1092

[B37] Kelstrup H. C. Hartfelder K. Esterhuizen N. Wossler T. C. (2017). Juvenile hormone titers, ovarian status and epicuticular hydrocarbons in gynes and workers of the paper wasp Belonogaster longitarsus. *J. Insect Physiol.* 98 83–92. 10.1016/j.jinsphys.2016.11.014 27913150

[B38] Kelstrup H. C. Hartfelder K. Nascimento F. S. Riddiford L. M. (2014). The role of juvenile hormone in dominance behavior, reproduction and cuticular pheromone signaling in the caste-flexible epiponine wasp, *Synoeca surinama*. *Front. Zool.* 11:78. 10.1186/s12983-014-0078-5 25371699 PMC4219083

[B39] Kikuchi T. Tsuji K. Ohnishi H. Le Breton J. (2007). Caste-biased acceptance of non-nestmates in a polygynous ponerine ant. *Anim. Behav.* 73 559–565. 10.1016/j.anbehav.2006.04.015

[B40] Kocher S. D. Grozinger C. M. (2011). Cooperation, conflict, and the evolution of queen pheromones. *J. Chem. Ecol.* 37 1263–1275. 10.1007/s10886-011-0036-z 22083225

[B41] Kohlmeier P. Feldmeyer B. Foitzik S. (2018). Vitellogenin-like A-associated shifts in social cue responsiveness regulate behavioral task specialization in an ant. *PLoS Biol.* 16:e2005747. 10.1371/journal.pbio.2005747 29874231 PMC5991380

[B42] Korb J. Weil T. Hoffmann K. Foster K. R. Rehli M. (2009). A gene necessary for reproductive suppression in termites. *Science* 324 758–758. 10.1126/science.1170660 19423819

[B43] Le Conte Y. Hefetz A. (2008). Primer pheromones in social hymenoptera. *Annu. Rev. Entomol.* 53 523–542. 10.1146/annurev.ento.52.110405.091434 17877458

[B44] Leonhardt S. D. Menzel F. Nehring V. Schmitt T. (2016). Ecology and evolution of communication in social insects. *Cell* 164 1277–1287. 10.1016/j.cell.2016.01.035 26967293

[B45] Liebig J. (2010). Hydrocarbon profiles indicate fertility and dominance status in ant, bee, and wasp colonies. *Insect Hydrocarb.* 2010 254–281. 10.1017/CBO9780511711909.014

[B46] Maisonnasse A. Alaux C. Beslay D. Crauser D. Gines C. Plettner E. (2010). New insights into honey bee (*Apis mellifera*) pheromone communication. Is the queen mandibular pheromone alone in colony regulation? *Front. Zool.* 7:18. 10.1186/1742-9994-7-18 20565874 PMC2897789

[B47] Manfredini F. Lucas C. Nicolas M. Keller L. Shoemaker D. Grozinger C. M. (2014). Molecular and social regulation of worker division of labour in fire ants. *Mol. Ecol.* 23 660–672. 10.1111/mec.12626 24329612

[B48] Matsuura K. Himuro C. Yokoi T. Yamamoto Y. Vargo E. L. Keller L. (2010). Identification of a pheromone regulating caste differentiation in termites. *Proc. Natl. Acad. Sci. U.S.A.* 107 12963–12968. 10.1073/pnas.1004675107 20615972 PMC2919916

[B49] McKenzie S. K. Fetter-Pruneda I. Ruta V. Kronauer D. J. (2016). Transcriptomics and neuroanatomy of the clonal raider ant implicate an expanded clade of odorant receptors in chemical communication. *Proc. Natl. Acad. Sci. U.S.A.* 113 14091–14096. 10.1073/pnas.1610800113 27911792 PMC5150400

[B50] Metcalf R. A. Whitt G. S. (1977). Intra-nest relatedness in the social wasp *Polistes metricus*. *Behav. Ecol. Sociobiol.* 2 339–351. 10.1007/BF00299504

[B51] Mohammedi A. Paris A. Crauser D. Le Conte Y. (1998). Effect of aliphatic esters on ovary development of queenless bees (*Apis mellifera* L.). *Naturwissenschaften* 85 455–458. 10.1007/s001140050531

[B52] Mutti N. S. Dolezal A. G. Wolschin F. Mutti J. S. Gill K. S. Amdam G. V. (2011). IRS and TOR nutrient-signaling pathways act via juvenile hormone to influence honey bee caste fate. *J. Exp. Biol.* 214 3977–3984. 10.1242/jeb.061499 22071189 PMC3212421

[B53] Nakanishi A. Nishino H. Watanabe H. Yokohari F. Nishikawa M. (2010). Sex-specific antennal sensory system in the ant *Camponotus japonicus*: glomerular organizations of antennal lobes. *J. Comp. Neurol.* 518 2186–2201. 10.1002/cne.22326 20437523

[B54] Naumann K. Winston M. L. Slessor K. N. Prestwich G. D. Webster F. X. (1991). Production and transmission of honey bee queen (*Apis mellifera* L.) mandibular gland pheromone. *Behav. Ecol. Sociobiol.* 29 321–332. 10.1007/bf00165956

[B55] Oi C. A. Oliveira R. C. van Zweden J. S. Mateus S. Millar J. G. Nascimento F. S. (2019). Do primitively eusocial wasps use queen pheromones to regulate reproduction? a case study of the paper wasp *Polistes satan*. *Front. Ecol. Evol.* 7:199 10.3389/fevo.2019.00199

[B56] Oi C. A. van Zweden J. S. Oliveira R. C. Van Oystaeyen A. Nascimento F. S. Wenseleers T. (2015). The origin and evolution of social insect queen pheromones: novel hypotheses and outstanding problems. *Bioessays* 37 808–821. 10.1002/bies.201400180 25916998

[B57] Oliveira R. C. Vollet-Neto A. Akemi Oi C. van Zweden J. S. Nascimento F. Sullivan Brent C. (2017). Hormonal pleiotropy helps maintain queen signal honesty in a highly eusocial wasp. *Sci. Rep.* 7 1–12. 10.1038/s41598-017-01794-1 28490760 PMC5431770

[B58] Orlova M. Amsalem E. (2019). Context matters: plasticity in response to pheromones regulating reproduction and collective behavior in social Hymenoptera. *Curr. Opin. Insect Sci.* 35 69–76. 10.1016/j.cois.2019.07.004 31404906

[B59] Orlova M. Hefetz A. (2014). Distance from the queen affects workers’ selfish behaviour in the honeybee (*A. mellifera*) colony. *Behav. Ecol. Sociobiol.* 68 1693–1700. 10.1007/s00265-014-1777-9

[B60] Orlova M. Starkey J. Amsalem E. (2020). A small family business: synergistic and additive effects of the queen and the brood on worker reproduction in a primitively eusocial bee. *J. Exp. Biol.* 223:547. 10.1242/jeb.217547 31953359

[B61] Oxley P. R. Ji L. Fetter-Pruneda I. McKenzie S. K. Li C. Hu H. (2014). The genome of the clonal raider ant *Cerapachys biroi*. *Curr. Biol.* 24 451–458. 10.1016/j.cub.2014.01.018 24508170 PMC3961065

[B62] Ozaki M. Wada-Katsumata A. Fujikawa K. Iwasaki M. Yokohari F. Satoji Y. (2005). Ant nestmate and non-nestmate discrimination by a chemosensory sensillum. *Science* 309 311–314. 10.1126/science.1105244 15947139

[B63] Padilla M. Amsalem E. Altman N. Hefetz A. Grozinger C. M. (2016). Chemical communication is not sufficient to explain reproductive inhibition in the bumblebee *Bombus impatiens*. *R. Soc. Open Sci.* 3:160576. 10.1098/rsos.160576 27853577 PMC5099002

[B64] Page R. E. Jr. Amdam G. V. (2007). The making of a social insect: developmental architectures of social design. *Bioessays* 29 334–343. 10.1002/bies.20549 17373656 PMC2398704

[B65] Pamminger T. Hughes W. O. (2017). Testing the reproductive groundplan hypothesis in ants (Hymenoptera: Formicidae). *Evolution* 71 153–159. 10.1111/evo.13105 27783387

[B66] Pandey A. Bloch G. (2015). Juvenile hormone and ecdysteroids as major regulators of brain and behavior in bees. *Curr. Opin. Insect. Sci.* 12 26–37. 10.1016/j.cois.2015.09.006

[B67] Pask G. M. Slone J. D. Millar J. G. Das P. Moreira J. A. Zhou X. (2017). Specialized odorant receptors in social insects that detect cuticular hydrocarbon cues and candidate pheromones. *Nat. Commun.* 8:297. 10.1038/s41467-017-00099-1 28819196 PMC5561057

[B68] Peeters C. Liebig J. (2009). “Fertility signaling as a general mechanism of regulating reproductive division of labor in ants,” in *Organization of Insect Societies: From Genome to Socio-Complexity*, eds Gadau J. Fewell J. (Cambridge: Harvard University Press), 220–242.

[B69] Peso M. Elgar M. A. Barron A. B. (2015). Pheromonal control: reconciling physiological mechanism with signalling theory. *Biol. Rev.* 90 542–559. 10.1111/brv.12123 24925630

[B70] Peter A. (2002). “Oviposition pheromones in herbivorous and carnivorous insects,” in *Chemoecology of Insect Eggs and Egg Depositions*, eds Hilker M. Meiners T. (Oxford: Blackwell Publishing), 235–263. 10.1002/9780470760253.ch9

[B71] Princen S. A. Van Oystaeyen A. Petit C. van Zweden J. S. Wenseleers T. Smiseth P. (2019). Cross-activity of honeybee queen mandibular pheromone in bumblebees provides evidence for sensory exploitation. *Behav. Ecol.* 31 303–310. 10.1093/beheco/arz191 27193460

[B72] Reichert M. S. Quinn J. L. (2017). Cognition in contests: mechanisms, ecology, and evolution. *Trends Ecol. Evol.* 32 773–785. 10.1016/j.tree.2017.07.003 28823589

[B73] Schultner E. Oettler J. Helantera H. (2017). The role of brood in eusocial Hymenoptera. *Q. Rev. Biol.* 92 39–78. 10.1086/690840 29558609

[B74] Sharma K. R. Enzmann B. L. Schmidt Y. Moore D. Jones G. R. Parker J. (2015). Cuticular hydrocarbon pheromones for social behavior and their coding in the ant antenna. *Cell Rep.* 12 1261–1271. 10.1016/j.celrep.2015.07.031 26279569

[B75] Sledge M. F. Boscaro F. Turillazzi S. (2001). Cuticular hydrocarbons and reproductive status in the social wasp *Polistes dominulus*. *Behav. Ecol. Sociobiol.* 49 401–409. 10.1007/s002650000311

[B76] Slessor K. N. Kaminski L. A. King G. G. S. Borden J. H. Winston M. L. (1988). Semiochemical basis of the retinue response to queen honey bees. *Nature* 332 354–356. 10.1038/332354a0

[B77] Smith A. A. Holldober B. Liebig J. (2009). Cuticular hydrocarbons reliably identify cheaters and allow enforcement of altruism in a social insect. *Curr. Biol.* 19 78–81. 10.1016/j.cub.2008.11.059 19135369

[B78] Smith A. A. Holldobler B. Liebig J. (2012). Queen-specific signals and worker punishment in the ant *Aphaenogaster cockerelli*: the role of the Dufour’s gland. *Anim. Behav.* 83 587–593. 10.1016/j.anbehav.2011.12.024

[B79] Smith A. A. Liebig J. (2017). The evolution of cuticular fertility signals in eusocial insects. *Curr. Opin. Insect Sci.* 22 79–84. 10.1016/j.cois.2017.05.017 28805643

[B80] Smith C. D. Zimin A. Holt C. Abouheif E. Benton R. Cash E. (2011). Draft genome of the globally widespread and invasive Argentine ant (*Linepithema humile*). *Proc. Natl. Acad. Sci. U.S.A.* 108 5673–5678. 10.1073/pnas.1008617108 21282631 PMC3078359

[B81] Soroker V. Vienne C. Hefetz A. (1995). Hydrocarbon dynamics within and between nestmates in *Cataglyphis niger* (Hymenoptera: Formicidae). *J. Chem. Ecol.* 21 365–378. 10.1007/bf02036724 24234067

[B82] Stabentheiner A. Kovac H. Brodschneider R. (2010). Honeybee colony thermoregulation - regulatory mechanisms and contribution of individuals in dependence on age, location and thermal stress. *PLoS One* 5:e8967. 10.1371/journal.pone.0008967 20126462 PMC2813292

[B83] Starkey J. Brown A. Amsalem E. (2019a). The road to sociality: brood regulation of worker reproduction in the simple eusocial bee *Bombus impatiens*. *Anim. Behav.* 154 57–65. 10.1016/j.anbehav.2019.06.004

[B84] Starkey J. Derstine N. Amsalem E. (2019b). Do bumble bees produce brood pheromones? *J. Chem. Ecol.* 45 725–734. 10.1007/s10886-019-01101-4 31471873

[B85] Steitz I. Ayasse M. (2020). Macrocyclic lactones act as a queen pheromone in a primitively eusocial sweat bee. *Curr. Biol.* 30 1136–1141. 10.1016/j.cub.2020.01.026 32059770

[B86] Stokl J. Steiger S. (2017). Evolutionary origin of insect pheromones. *Curr. Opin. Insect Sci.* 24 36–42. 10.1016/j.cois.2017.09.004 29208221

[B87] Tibbetts E. A. Fearon M. L. Wong E. Huang Z. Y. Tinghitella R. M. (2018). Rapid juvenile hormone downregulation in subordinate wasp queens facilitates stable cooperation. *Proc. R. Soc. B* 285:2645. 10.1098/rspb.2017.2645 29436498 PMC5829203

[B88] Tibbetts E. A. Lindsay R. (2008). Visual signals of status and rival assessment in *Polistes dominulus* paper wasps. *Biol. Lett.* 4 237–239. 10.1098/rsbl.2008.0048 18331973 PMC2610050

[B89] Ulrich Y. Burns D. Libbrecht R. Kronauer D. J. (2016). Ant larvae regulate worker foraging behavior and ovarian activity in a dose-dependent manner. *Behav. Ecol. Sociobiol.* 70 1011–1018. 10.1007/s00265-015-2046-2 27616809 PMC5015688

[B90] Van Oystaeyen A. Oliveira R. C. Holman L. van Zweden J. S. Romero C. Oi C. A. (2014). Conserved class of queen pheromones stops social insect workers from reproducing. *Science* 343 287–290. 10.1126/science.1244899 24436417

[B91] Velasque M. Qiu L. Mikheyev A. S. (2018). The doublesex sex determination pathway regulates reproductive division of labor in honey bees. *bioRxiv* [Preprint], 10.1101/314492

[B92] Vergoz V. McQuillan H. J. Geddes L. H. Pullar K. Nicholson B. J. Paulin M. G. (2009). Peripheral modulation of worker bee responses to queen mandibular pheromone. *Proc. Natl. Acad. Sci. U.S.A.* 106 20930–20935. 10.1073/pnas.0907563106 19934051 PMC2791564

[B93] Villalta I. Abril S. Cerda X. Boulay R. (2018). Queen control or queen signal in ants: what remains of the controversy 25 years after Keller and Nonacs’ seminal paper? *J. Chem. Ecol.* 44 805–817. 10.1007/s10886-018-0974-9 29858748

[B94] Wanner K. W. Nichols A. S. Walden K. K. Brockmann A. Luetje C. W. Robertson H. M. (2007). A honey bee odorant receptor for the queen substance 9-oxo-2-decenoic acid. *Proc. Natl. Acad. Sci. U.S.A.* 104 14383–14388. 10.1073/pnas.0705459104 17761794 PMC1964862

[B95] West-Eberhard M. J. (1987). “Flexible strategy and social evolution,” in *Animal Societies. Theories and Facts*, eds Ito Y. Brown J. L. Kikkawa J. (Tokyo: Japan Scientific Societies Press), 35–51.

[B96] Wilson E. O. (1971). *The Insect Societies.* Cambridge: Harvard University Press.

[B97] Wyatt T. D. (2014). *Pheromones and Animal Behavior: Chemical Signals and Signatures.* Cambridge: Cambridge University Press.

[B98] Yan H. Opachaloemphan C. Mancini G. Yang H. Gallitto M. Mlejnek J. (2017). An engineered orco mutation produces aberrant social behavior and defective neural development in ants. *Cell* 170 736–747. 10.1016/j.cell.2017.06.051 28802043 PMC5587193

[B99] Zhou X. Slone J. D. Rokas A. Berger S. L. Liebig J. Ray A. (2012). Phylogenetic and transcriptomic analysis of chemosensory receptors in a pair of divergent ant species reveals sex-specific signatures of odor coding. *PLoS Genet.* 8:e1002930. 10.1371/journal.pgen.1002930 22952454 PMC3431598

